# Birth outcomes, pregnancy complications, and postpartum mental health after the 2013 Calgary flood: A difference in difference analysis

**DOI:** 10.1371/journal.pone.0246670

**Published:** 2021-02-11

**Authors:** Erin Hetherington, Kamala Adhikari, Lianne Tomfohr-Madsen, Scott Patten, Amy Metcalfe

**Affiliations:** 1 Department of Obstetrics & Gynaecology, Cumming School of Medicine, University of Calgary, Calgary, Alberta, Canada; 2 Department of Community Health Sciences, Cumming School of Medicine, University of Calgary, Calgary, Alberta, Canada; 3 Alberta Cancer Prevention Legacy Fund, Alberta Health Services, Calgary, Alberta, Canada; 4 Department of Psychology, Faculty of Arts, University of Calgary, Calgary, Alberta, Canada; 5 Department of Psychiatry, Cumming School of Medicine, University of Calgary, Calgary, Alberta, Canada; Norwegian Institute of Public Health, NORWAY

## Abstract

**Background:**

In June 2013, the city of Calgary, Alberta and surrounding areas sustained significant flooding which resulted in large scale evacuations and closure of businesses and schools. Floods can increase stress which may negatively impact perinatal outcomes and mental health, but previous research is inconsistent. The objectives of this study are to examine the impact of the flood on pregnancy health, birth outcomes and postpartum mental health.

**Methods:**

Linked administrative data from the province of Alberta were used. Outcomes included preterm birth, small for gestational age, a new diagnoses of preeclampsia or gestational hypertension, and a diagnosis of, or drug prescription for, depression or anxiety. Data were analyzed using a quasi-experimental difference in difference design, comparing flooded and non-flooded areas and in affected and unaffected time periods. Multivariable log binomial regression models were used to estimate risk ratios, adjusted for maternal age. Marginal probabilities for the difference in difference term were used to show the potential effect of the flood.

**Results:**

Participants included 18,266 nulliparous women for the pregnancy outcomes, and 26,956 women with infants for the mental health analysis. There were no effects for preterm birth (DID 0.00, CI: -0.02, 0.02), small for gestational age (DID 0.00, CI: -0.02, 0.02), or new cases of preeclampsia (DID 0.00, CI: -0.01, 0.01). There was a small increase in new cases of gestational hypertension (DID 0.02, CI: 0.01, 0.03) in flood affected areas. There were no differences in postpartum anxiety or depression prescriptions or diagnoses.

**Conclusion:**

The Calgary 2013 flood was associated with a minor increase in gestational hypertension and not other health outcomes. Universal prenatal care and magnitude of the disaster may have minimized impacts of the flood on pregnant women.

## Background

Floods are the most common type of natural disaster causing significant health and financial impacts [1–3]. Extreme weather events are expected to increase due to climate change, and these events will have consequences for both physical and mental health [4]. In June 2013, major flooding of over 55,000 square kilometers in southern Alberta, Canada led to the evacuation of over 100,000 residents in the region and damaging 14,500 homes [5, 6]. Areas highly impacted by flooding included Calgary, a major urban center (pop. 1.2 million), and smaller cities including High River and Canmore (pop. 13,000 each) [5]. In Calgary, the energy industry center for Canada, the central business district was closed for over a week, and there were disruptions to schools and utilities. The damage from the flood was estimated at over 6 billion Canadian dollars, one of the most expensive natural disasters in Canadian history [6].

Natural disasters can have serious mental and physical health consequences. In addition to the physical threats caused by injury or exposure to toxins or contamination, natural disasters can have important mental health consequences. The impact of natural disasters can be difficult to quantify due to the varied nature and intensity of extreme events, as well as the differences in the social and physical infrastructures in communities they affect [4]. Natural disasters are by their definition unpredictable, unexpected, and uncontrollable events which result in a collective stress response in the impacted communities [7]. However, individualized responses to mass events will differ depending on pre-existing vulnerabilities and coping resources [8, 9]. At least a portion of those impacted can be expected to develop psychopathological responses including post-traumatic stress disorder, anxiety, or depression following natural disasters [10–12].

Elevated stress can have several health impacts on birth outcomes including preterm birth and low birthweight, pregnancy health, including gestational hypertension and preeclampsia, and mental health symptoms [13–17]. However, the literature linking natural disasters to birth outcomes is mixed. Several smaller convenience sample studies have shown increases in preterm birth or low birthweight associated with natural disasters including hurricane Katrina and several earthquakes [18–20]. Similarly, two hospital based studies of earthquakes in China and Chile and showed worse birth outcomes (including preterm birth and low birthweight) using pre and post disaster designs [21, 22]. However, these smaller studies may have suffered from selection bias due to how they identified participants. Larger studies using vital statistics and administrative records tend to show no differences in birth outcomes [23–25]. For example, Sugawara and colleagues compared incidence of low birthweight and preterm birth in coastal regions to inland regions in Japan after a tsunami in 2011 and found no differences, however this study was not able to account for residual confounding due to regional differences [25]. On the other hand, Tong et al. found increases in low birthweight and preterm birth after major flooding from the North Dakota Red River floods of 1997, and Antipova found increases in preterm birth after hurricane Andrew in Florida, both studies used a pre-post design [26, 27]. However, demographic changes in the pregnant population over the study period could have resulted in residual confounding [27]. Some studies have also examined the impact of timing of exposure, mostly noting that exposure earlier in pregnancy may result in more adverse outcomes [18, 28–30]. Research among women who were pregnant during a catastrophic ice storm in Quebec, Canada showed higher levels of maternal stress were associated with infants of shorter birth length, and effects were stronger for first trimester exposure as well as for male infants [29]. Research on earthquakes in Chile and the United States suggest that exposure during the first trimester may shorten gestational length, particularly among females [18, 28].

Fewer studies have examined the impact of natural disasters on pregnancy related health conditions such as gestational hypertension and preeclampsia, which may also be impacted by stress [31, 32]. Research from both the United States and Japan have found elevated preeclampsia or gestational hypertension, following natural disasters (hurricanes, floods, earthquakes), but these analyses could be sensitive to regional differences or time trends [27, 33–35].

Finally, there has been a significant amount of literature examining the impact of natural disasters on mental health showing increases in post-traumatic stress disorder, anxiety and depression [10, 12]. The pregnancy specific literature tends to show higher rates of post-traumatic stress disorder and depression [20, 36, 37]. However, these studies are small cross-sectional studies without adequate comparison groups.

Studying health outcomes after natural disasters is challenging. Participant recruitment can be difficult because of the disruption caused by the disaster and competing priorities on safety and rebuilding. Specifically recruiting of participants may result in significant selection bias, and challenges in obtaining an adequate control group [10]. Relying on administrative or routinely collected data can improve sample size, but quantifying exposure, and obtaining an appropriate control group remains challenging. Most studies using routinely collected data use geographic location as a measure of exposure, which may result in more people that are indirectly exposed being included in the study, thus diluting the measure of exposure [10, 38]. Even more problematic is the choice of an appropriate control group. Because natural disasters are unpredictable, some studies rely on cross-sectional data, using regional comparisons (e.g. coastal vs inland residents for hurricane exposure). However, this study design cannot account for baseline differences between groups living in different areas, and may result in unmeasured confounding [10, 38]. Pre-post designs are also common, but these cannot account for temporal trends due to demographic shifts in the population [10, 27, 38].

**Fig 1:**
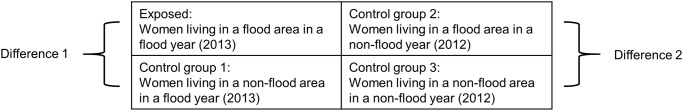
Conceptual framework for a difference in difference analysis.

A difference in difference (DID) study design attempts to overcome challenges associated with regional comparisons and pre-post designs. The DID design is a quasi-experimental research design used to study causal relationships in observational studies. A simple case of a DID is two different groups in two different time periods [39]. Using the example of the 2013 Calgary flood would correspond to women living in a flood area during the year of the flood (exposure group), women in a non-flood area during the year of the flood (control group 1), women living in a flood area in a non-flood year (control group 2) and women living in a non-flood area in a non-flood year (control group 3). See Fig 1. Difference 1 examines the impact of living in a flood area vs. not during a flood year. Difference 2 is the same geographical comparator in a non-flood year. Together, these two differences should account for any unmeasured confounding between the two geographical areas, as long as this unmeasured confounding does not change over time [39]. The difference between difference 1 and difference 2 (DID) examines the impact of time (having already accounted for geography).

The aims of this research study are: 1) to examine the impact of the 2013 Calgary flood on birth outcomes (preterm birth and small for gestational age) and pregnancy health (gestational hypertension and preeclampsia) and 2) to examine the impact of the 2013 Calgary flood on mental health outcomes (anxiety and depression).

## Methods

### Data sources

A secondary data analysis was conducted on administrative data from 5 databases containing health information of residents of Alberta Canada with data from 2012 to 2015. The Alberta Perinatal Health Program dataset contains information on all live and stillbirths in the province that occurred in a hospital or were attended by a registered midwife at home or a birthing center. Information includes delivery characteristics and newborn health including gestational age at delivery, birthweight as well as limited demographic information such as maternal age and postal code. The Discharge Abstract Database (DAD) contains administrative, clinical and demographic information on hospital discharges. Data include up to 25 diagnoses associated with each hospitalization coded according to International Classification of Diseases, Tenth Revision, Canada (ICD-10- CA) coding. The National Ambulatory Care Reporting System (NACRS) contains hospital-based and community-based ambulatory care from day surgeries, outpatient and hospital-based clinics and emergency departments. NACRS contains up to 10 diagnoses for each encounter. Data are abstracted by trained personnel using standardized procedures and definitions provided by the Canadian Institute for Health Information. Physician Claims contains clinical information submitted by practitioners for billing claims, including up to three diagnoses coded according to ICD-9-CM coding (ninth version, clinical modification). The Pharmaceutical Information Network (PIN) contains information on dispensations of prescriptions at community pharmacies. PIN contains prescription information including dosage and active ingredients classified according to the Anatomical Therapeutic Chemical (ATC) coding system. These databases were originally created for healthcare management and monitoring (e.g., insurance claims and remunerating physicians) under the universal healthcare system in Canada. These data holdings in Alberta are maintained by Alberta Health and Alberta Health Services. Deterministic linkage using personal health numbers was used to link data across datasets. These data cover over 99% of the general population of the province and are considered high quality and suitable for research purposes [40]. This study received ethical approval from the University of Calgary Conjoint Health Research Ethics Board (REB 17–0430). A waiver of consent was obtained for this analysis of anonymized administrative data.

### Study population

Two study cohorts were identified, the first for pregnancy related health and outcomes (pregnancy health cohort) and the second for mental health outcomes (mental health cohort). Inclusion criteria for the pregnancy health cohort were based on timing of pregnancy, geography and parity. Women had to be pregnant at the time of the flood (June 19, 2013) or one year prior to the flood. Women had to be living in a flood area or control area as determined by postal code. The flood area included all communities of Calgary, Canmore, and High River which were highly impacted by flooding. The control area included Edmonton, Whitecourt, and Wetaskiwin, communities that were geographically similar to flooded areas in size terms of size and distance from a major urban center. Because many women had more than one pregnancy during the time period, only nulliparous women were included. This was done to minimize possible bias due to parity. Women with pregnancy complications (e.g. preterm birth or gestational hypertension) are more likely to have similar complications in a subsequent pregnancy. Because the control period was one year prior to the exposure period, a women’s earlier pregnancy might have been included in the control year, but then a subsequent pregnancy excluded in the exposure year (to ensure women had only been included once in analyses). This could artificially inflate pregnancy complications in the control year.

Inclusion criteria for the mental health cohort was based on postpartum period, timing and geography. Women had to have an infant under the age of 6 months at the time of the flood, or one year after, and live in the flood or control areas as defined above. We then followed up these women for 6 months to identify prescriptions or diagnoses after the flood. This strategy allowed for a fixed follow-up time for women but allowed for the inclusion of women with infants up to one year of age. The control year was set at 2014 instead of 2012 because the dataset only included births as of April 2012, which meant not all infants under the age of 6 months in June 2012 would be included. The focus or the mental health analysis was on women with young infants because many women in pregnancy choose not to take antidepressant or anxiolytic prescriptions during pregnancy [41].

### Outcomes

For the pregnancy health study, outcomes included preterm birth, small for gestational age, a new diagnosis of preeclampsia, or a new diagnosis of hypertension. Preterm birth was defined as a live birth before 37 weeks gestation and was obtained from the APHP. Pregnancies greater or equal to 37 weeks at the index date (June 19) were excluded from the preterm birth analysis to prevent immortal time bias. Small for gestational (SGA) age was calculated using Canadian growth reference standards with infants below the 10th percentile for gestational age being considered SGA [42]. Preeclampsia and gestational hypertension cases were identified in the DAD and NACRS using validated ICD-10-CA codes, and in Physician Claims using ICD-9-CM codes validated in a Canadian context [43]. A list of codes is available in S1 Appendix. These codes show high levels of sensitivity and specificity (e.g. 87.9% and 99.6% for gestational hypertension) in Canadian administrative data [44]. Only new diagnoses after the index date (June 19, 2013 for the exposure year, and June 19, 2012 for the control year) were included. That is to say, if a woman had a diagnosis of preeclampsia on June 4, 2013 and again on June 25, 2013, this was not considered a “new case” and was not included.

For the mental health study, any diagnosis of any depressive or anxiety disorder or prescription for an antidepressant or anxiolytic for six months after the index date (June 19) was included. Because mental health conditions are often cyclical, and can be exacerbated by external events, we chose to include any diagnosis or prescription after the index date, not just a new diagnosis. We used broad definitions using ICD-10-CA and ICD-9-CM coding for diagnoses and high-level classifications of antidepressants and anxiolytics from the Anatomical Therapeutic Chemical (ATC) Classification system [45, 46]. (see S1 Appendix for all codes). We chose broad definitions because mental health conditions can often co-occur and because broader definitions increase sensitivity with minimal loss of specificity [45–47]. Validation studies comparing these codes to medical charts have reported sensitivity of 63.5% and specificity of 86.8% for mood and anxiety disorders [45].

### Analytic strategy

We used a difference in difference analytical approach. For the pregnancy health study, the exposed group was women who were pregnant and living in a flood area on June 19, 2013. The control groups were: 1) women who were pregnant and living in a control area on June 19, 2013; 2) women who were pregnant and living in a flood area on June 19, 2012; and 4) women who were pregnant and living in control area on June 19, 2012. For the mental health study, a similar strategy was used, but instead of pregnant women, it was women with a child less than 6 months and the control year was 2014 instead of 2012 (one year after the flood year). We estimated risk ratios using log-binomial regression, adjusted for maternal age using the following formula: log(p) = ß0 + ß1 Year + ß2 Area +ß3 YearArea + ß4 Maternal Age. Year was coded as 1 for the flood year and 0 for the control year. Area was coded as 1 for the flood area, and 0 for the control area. YearArea was a product term between year and area, and is the difference in difference estimator. Maternal age at delivery was continuous. If ß3 was statistically significant, that indicated that the difference in log prevalence between the flood and control areas, also differ by year. After fitting the regression equation for each outcome, we estimated the marginal probabilities for the difference in difference term to show the potential effect of the flood. We then plotted marginal predicted probabilities graphically to illustrated differences. We conducted a number of sensitivity analyses including repeating the main regression analysis stratified by trimester of pregnancy at the time of the flood for pregnancy health outcomes, sex of the infant for all outcomes, and parity for mental health outcomes. Finally, we repeated all analyses restricting the exposure level to neighbourhoods that were under direct evacuation orders in Calgary (along the river valley and of mixed socio-demographic composition) compared to geographically and economically similar neighbourhoods in the control area.

## Results

The APHP dataset contained 159,059 births from 2012 to 2013. Of these, 865 had incomplete personal health numbers (574), or could not be successfully linked to any of the other databases (291) and were eliminated. For the pregnancy health population, 18,291 nulliparous women were identified (being pregnant on June 19, 2013, or one year prior, and living in a flood or control area). There was minimal change in population from one year to the next (<2%) suggesting minimal shifts in the underlying source population. For the mental health population, 26,956 women were identified (having a baby less than 6 months of age on June 19, 2013, or one year after, and living in a flood or control area). Descriptive statistics for the overall sample, and broken down by year and geography, can be found in Table 1.

**Table 1:** Sample characteristics.

Pregnancy Outcomes	Overall		2012				2013			
	n = 18291		Control n = 3945		Flood n = 5089		Control n = 4077		Flood n = 5180	
	n	%	n	%	n	%	n	%	n	%
Maternal age (mean, sd)	28.9	5.2	28.0	5.2	29.4	5.2	28.2	5.2	29.8	4.9
Preterm birth*	1455	8.4	3730	8.4	4788	7.8	3841	9.2	4865	8.6
Small for gestational age	2696	14.7	500	12.7	788	15.5	560	13.7	848	16.4
Preeclampsia	854	4.7	188	4.8	245	4.8	184	4.5	237	4.6
Gestational hypertension	1098	6.0	235	6.0	296	5.8	204	5.0	363	7.0
Mental Health Outcomes	Overall		2014				2013			
	n = 26956		Control n = 6188		Flood n = 7641		Control n = 5788		Flood n = 7339	
	n	%	n	%	n	%	n	%	n	%
Maternal age (mean, sd)	30.5	5.2	30.1	5.2	31.2	5.0	29.8	5.3	30.9	5.1
Diagnosis or prescription for anxiety or depression	2690	10.0	604	9.8	742	9.7	606	10.5	738	10.1

*The overall n for preterm birth is n = 17224, 1067 women were excluded from the analysis because they were more than 37 weeks gestation at the time of the flood and could not have a preterm delivery.

**Fig 2:**
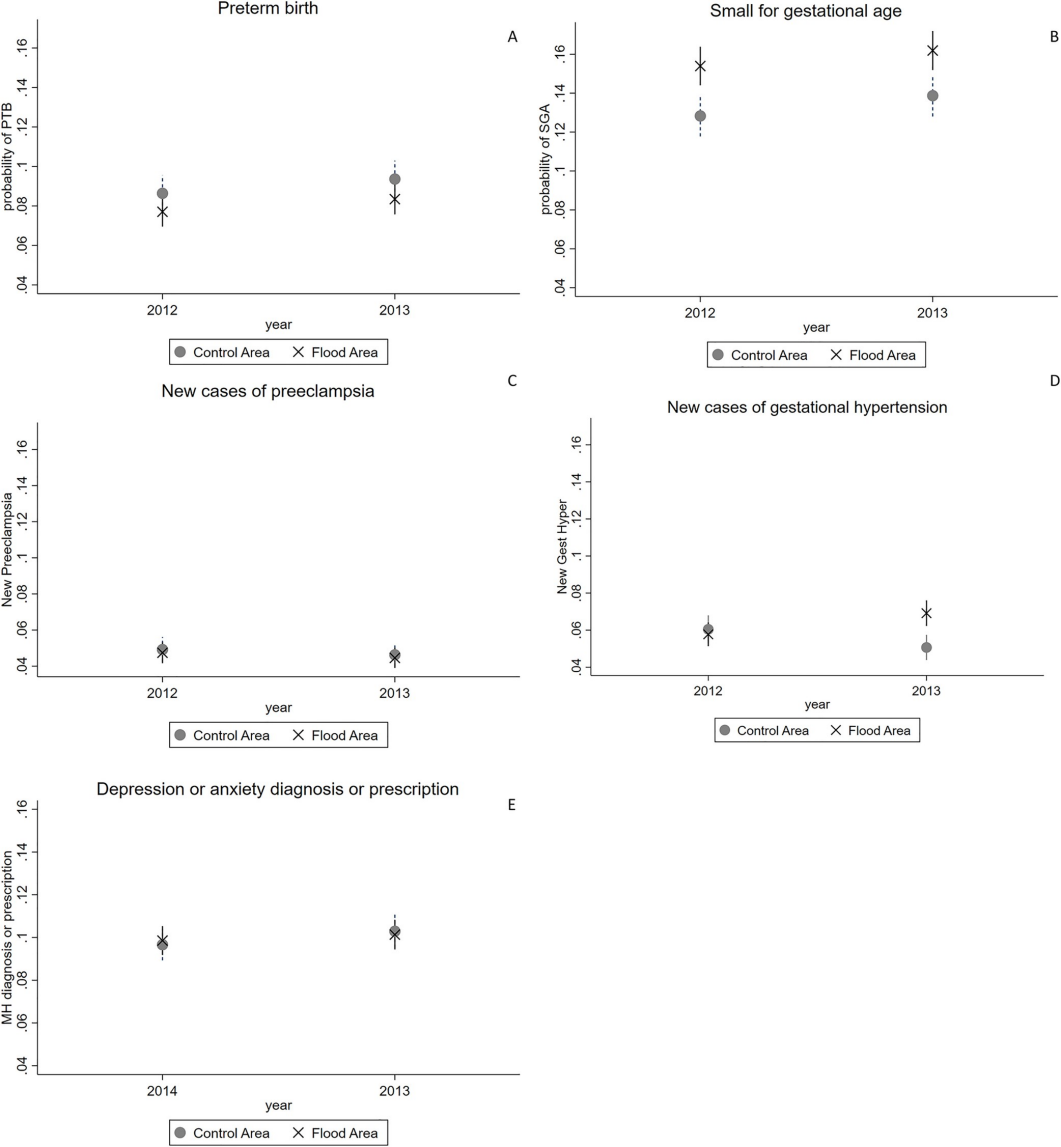
Marginal probabilities of outcomes. Panel A: marginal probabilities of preterm birth in flood and control areas in 2013 and 2012, n = 17224; Panel B: marginal probabilities of small for gestational age in flood and control areas in 2013 and 2012 n = 18291; Panel C: marginal probabilities of new case of preeclampsia in flood and control areas in 2013 and 2012 n = 18291; Panel D: marginal probabilities of new cases of gestational hypertension in flood and control areas in 2013 and 2012 n = 18291; Panel E: marginal probabilities of a diagnosis or prescription for anxiety or depression in flood and control areas in 2013 and 2014 n = 26956.

Table 2 shows the marginal effects and 95% CI for the difference in difference estimator. There were no differences in preterm birth, small for gestational age, preeclampsia or mental health outcomes. There was a very small difference in gestational hypertension. Fig 2 shows differences graphically.

**Table 2:** Marginal effect of differences.

	Adjusted DID	(95% CI)
Pregnancy related outcomes		
Preterm birth	0.00	(-0.02, 0.02)
Small for gestational age	0.00	(-0.02, 0.02)
Preeclampsia	0.00	(-0.01, 0.01)
Gestational hypertension	0.02	(0.01, 0.03)
Mental health outcomes		
Diagnosis or prescription for depression or anxiety	0.00	(-0.02, 0.01)

Sensitivity analyses showed no difference in outcomes by trimester of pregnancy at the time of the flood, infant sex or parity for mental health outcomes. Results did not change when we restricted the analysis to women living in neighbourhoods with evacuation orders.

## Discussion

Our results show virtually no differences between pregnancy related outcomes and mental health outcomes attributable to the Calgary 2013 flood. The only exception was a very minor increased risk of developing gestation hypertension, which corresponds to a 2% increased marginal probability of gestational hypertension among those living in a flood area in 2013. The stress of living in a flood area may have contributed to increased cases of gestational hypertension, but the effect was minimal.

There were no differences in preterm birth, small for gestational age, or new cases of preeclampsia among women who were exposed to flooding. Our null findings for preterm birth and small for gestational age are consistent with a study of the impact of hurricanes in Florida on birth outcomes that also used a difference in difference design [24], but in contrast to other studies using pre-post designs [26, 27]. We found no impact of the flood on preeclampsia, which was in contrast to Tong et. al’s finding from the 1994 Red River flood [27]. The strength of our study lies in its analytic strategy which accounts for potential unmeasured confounding by both region and time period.

Our null results for preterm birth and small for gestational age may be partially explained by the presence of a robust healthcare system. In Canada, healthcare is universal, free, and fewer than 5% of women receive inadequate prenatal care [48, 49]. Comprehensive prenatal care may help buffer women from unexpected shocks caused by natural disasters, whereas in places where prenatal care is not universally available or is disrupted, disasters may exacerbate existing vulnerabilities in the population. A study of birth outcomes after the 2011 tsunami in Japan which found lower preterm birth and low birthweight in affected areas noted that a comprehensive prenatal care system and preventative medical intervention could help explain those finding [25]. On the other hand, some studies on birth outcomes after hurricanes in the United States have also found no impact on preterm birth, although there was less consistent evidence towards low birthweight [23, 33, 38].

Another factor that could contribute to our null findings is the magnitude of the disaster. Absolute measures of the impact of disasters are difficult to obtain because these will differ according to the magnitude of the event, but also of the underlying infrastructure, public services and response systems [10, 50]. The province of Alberta had a relatively coordinated disaster response, and despite the large economic impact, there were only 5 deaths because of the flood [6]. It is possible that the scale of this particular disaster was not large enough to significantly impact health outcomes.

The lack of differences in mental health outcomes was unexpected. Additional research using provincial administrative data from prescriptions showed an increase in anxiolytics in the 6 weeks following the flood [51]. Our sample population for this analysis did not include pregnant women, because many women are reluctant to take prescription medication during pregnancy. However, by including women with infants under the age of 6 months, we may also have included a population that was less inclined to take prescription medications while breastfeeding [52]. Approximately 87% of women in Canada initiate breastfeeding, and 26% are still exclusively breastfeeding at six months [53]. Mental health conditions are generally underrepresented in administrative data, and our algorithms likely did not capture all women with elevated depression or anxiety symptoms. Other Canadian research has indicated prevalence of postpartum depression at up to 15%, whereas in our administrative data, we only found 10% of women with either postpartum depression or anxiety [54]. Our estimates are unlikely to differ by exposure category, but would lead to an underestimation of mental health outcomes overall which could reduce our ability to detect differences in our DID analysis.

Our study did find a very small increase in cases in gestational hypertension that may be attributable to the flood. A study of the impact of Hurricane Sandy also found increases in gestational hypertension in ER visits after the storm [35]. Another study in Iceland found that gestational hypertension, but not preeclampsia, increased slightly after a major economic collapse in 2008 [55]. This suggests that the stress caused by major unexpected events may increase milder forms of hypertensive disorders (gestational hypertension), but it is not sufficient to cause a major hypertensive disorder such as preeclampsia.

### Limitations

The use of administrative data for research has inherent limitations. We were only able to ascertain residence at the time of birth, not at the time of exposure. It is therefore possible that women in flooded areas were displaced by the flood which could result in an underestimate of exposure. However, numbers of pregnancies in flood and non-flood years only differed by 2%, suggesting significant out-migration was unlikely. We were unable to control for variables such as income, education or ethnicity because these data were not available in our datasets. However, we did not expect the distribution of these variables to change markedly within the two geographical areas, so our difference in difference analysis should have been able to eliminate any unmeasured confounding due to these factors. It is possible that the impacts of the flood were more acutely experienced in more vulnerable individuals, which we were not able to examine. When using administrative data, there is always the chance of misclassification errors during data collection or abstraction. Moreover, for mental health outcomes, mental health diagnoses are not always captured in administrative data, and prescription data can only serve as a proxy for symptoms [46]. Our use of administrative data would also miss women who had mental health challenges but did not seek help from a physician, or sought help from a psychologist or other professional operating outside our administrative claims data. This would likely lead to an underassessment of mental health challenges, although this bias is likely non-differential, further biasing our results towards the null.

DID analyses rely on key assumptions, most importantly that unmeasured variables are consistent across groups or time [39]. This assumption is difficult to test, but there were no major healthcare or demographic changes between our exposed and control areas during the study period suggesting that any unmeasured confounding was likely consistent over time. DID analyses can be underpowered to detect small differences, which could also have contributed to our null findings [39].

Finally, in any disaster research, exposure to the disaster is often difficult to quantify. We chose to define exposure broadly as anyone living within the areas impacted by flooding. This is because while not all areas of metropolitan Calgary were flooded, the downtown core was impacted, which then impacted many businesses and people who might have worked in the downtown core. So while someone’s personal dwelling might not have sustained damage, they might have been unable to work, causing additional stress. Moreover, previous research on the Calgary flood indicated that mental health symptoms increased across the city regardless of direct impact on a dwelling, suggesting that the stress experienced by the flood was felt throughout the broadly impacted area [56].

## Conclusion

While the 2013 Calgary flood was a major natural disaster in Canada, it had no effect on preterm birth, small for gestational age, incidence of preeclampsia, or on postpartum mental health. The flood was associated with only a very minor increase in gestational hypertension. The magnitude of the disaster and coordinated disaster response could have mitigated negative outcomes. In addition, the availability of universal prenatal care in Canada could have minimized impacts of the flood on pregnant women.

## Supporting information

S1 Appendix (DOCX)
