# Supplementary material for: Birth outcomes, pregnancy complications, and postpartum mental health after the 2013 Calgary flood: A difference in difference analysis
Source: PLoS One. 2021 Feb 11;16(2):e0246670. doi: 10.1371/journal.pone.0246670 (PMC7877569; doi:10.1371/journal.pone.0246670)
Supplement: S1 Appendix — (DOCX) [file pone.0246670.s001.docx]

| Outcome | Codes used (unless specified, codes refer to higher level, and lower level codes are included) |
| --- | --- |
| Gestational Hypertension (without pre-eclampsia or pre-existing hypertension) | ICD-9-CM: 642.3  (without: 642.0, 642.1, 642.2, 642.4, 642.5, 642.7, 642.6, 401-405)  ICD-10-CA: O13, O16  (without: O10, O11, O14, O15, I10, I11, I12, I13, I15) |
| Preeclampsia | ICD-9-CM: 642.4 – 642.7  ICD-10-CA: O11, O14, O15 |
| Anxiety or depression diagnosis | ICD-9-CM: 296, 300, 311  ICD-10-CA: F30-39, F40-48, F68 |
| Anxiety or depression prescription | ATC: N05A, NO5B, NO6A |
